# Impact of hyper- and hypothermia on cellular and whole-body physiology

**DOI:** 10.1186/s40560-024-00774-8

**Published:** 2025-01-13

**Authors:** Toshiaki Iba, Yutaka Kondo, Cheryl L. Maier, Julie Helms, Ricard Ferrer, Jerrold H. Levy

**Affiliations:** 1 Department of Emergency and Disaster Medicine, Juntendo University Graduate School of Medicine, 2-1-1 Hongo Bunkyo-Ku, Tokyo, 113-8421 Japan; 2 Department of Emergency and Disaster Medicine, Juntendo University Graduate School of Medicine, Tokyo, Japan; 3 Department of Pathology and Laboratory Medicine, Emory University School of Medicine, Atlanta, GA USA; 4 Strasbourg University (UNISTRA), Strasbourg University Hospital, Medical Intensive Care Unit-NHC; INSERM (French National Institute of Health and Medical Research), UMR 1260, Regenerative Nanomedicine (RNM), FMTS, Strasbourg, France; 5 Intensive Care Department, Hospital Universitari Vall d’Hebron Universitat Autònoma de Barcelona, Barcelona, Spain; 6 Department of Anesthesiology, Critical Care, and Surgery, Duke University School of Medicine, Durham, NC USA

**Keywords:** Hyperthermia, Hypothermia, Inflammation, Coagulation, Cell death

## Abstract

The incidence of heat-related illnesses and heatstroke continues to rise amidst global warming. Hyperthermia triggers inflammation, coagulation, and progressive multiorgan dysfunction, and, at levels above 40 °C, can even lead to cell death. Blood cells, particularly granulocytes and platelets, are highly sensitive to heat, which promotes proinflammatory and procoagulant changes. Key factors in heatstroke pathophysiology involve mitochondrial thermal damage and excessive oxidative stress, which drive apoptosis and necrosis. While the kinetics of cellular damage from heat have been extensively studied, the mechanisms driving heat-induced organ damage and death are not yet fully understood. Converse to hyperthermia, hypothermia is generally protective, as seen in therapeutic hypothermia. However, accidental hypothermia presents another environmental threat due to arrhythmias, cardiac arrest, and coagulopathy. From a cellular physiology perspective, hypothermia generally supports mitochondrial homeostasis and enhances cell preservation, aiding whole-body recovery following resuscitation. This review summarizes recent findings on temperature-related cellular damage and preservation and suggests future research directions for understanding the tempo-physiologic axis.

## Introduction

Heat-related health emergencies have reached record-high levels in some parts of the world. In the United States, for example, the summer of 2023 saw unprecedented rates of heat-related illnesses. In July and August 2023, more than 300 out of every 100,000 emergency department visits were for heat-related illnesses, nearly 50% higher than the average peak rate from 2018 to 2022 [1].

Urbanization plays a significant role in exacerbating heat-related health risks. Built environments are commonly hotter than their neighboring rural areas, a phenomenon known as the urban heat island effect [2]. Urban heat islands effect can affect health directly and indirectly, with studies showing that the increased heat has a direct effect on mortality and morbidity, particularly during extreme heat events. A study of 13,115 urban settlements found that global exposure to extreme heat increased nearly 200% from 1983 to 2016. By 2100, heat-related mortality in Europe is expected to increase by about 50 times due to climate change, augmented by urban expansion [3].

The combination of climate change and the urban heat island effect is expected to amplify future heat effects. Previous studies report that dehydration, electrolyte imbalance, systemic hypotension, activated inflammation, and coagulation disturbances are involved in the pathophysiology of heatstroke [4]. More recently, various types of cell death induced by thermal injury have been shown to cause multiple organ dysfunction in heatstroke [5].

Hypothermia presents another health risk associated with low temperatures, with cardiac arrest being the primary cause of mortality in hypothermic patients. The risk of cardiac arrest sharply increases when core body temperature drops below 30 °C in young, healthy individuals and below 32 °C in older adults [6]. Each year, accidental hypothermia results in thousands of fatalities. However, due to the cytoprotective nature of hypothermia, full recovery can be achievable as long as organ injury, coagulopathy, and immune suppression are properly managed [7].

Since basic research in this area has been limited, here we summarize the current knowledge regarding cellular and body damage related to hyper- and hypothermia to illuminate potential areas of innovation in temperature-related illness research.

## Hyperthermia deteriorates mitochondrial function

Hyperthermia significantly impairs mitochondrial function by disrupting key cellular processes. For example, moderate hyperthermia around 40 °C increases the permeability of the mitochondrial inner membrane, leading to a loss of membrane potential and impaired oxidative phosphorylation. This effect can result in inefficient adenosine triphosphate (ATP) production, particularly at temperatures above 40 °C [8]. Elevated temperatures also increase reactive oxygen species (ROS) production within mitochondria, contributing to oxidative stress and cellular damage. ROS generated under hyperthermia can trigger apoptosis pathways [9]. Damage to the respiratory chain complexes is another mechanism of mitochondrial dysfunction [10]. Hyperthermia can specifically impair the function of electron transport chain complexes, with complex I being particularly vulnerable. This impairment reduces mitochondrial efficiency, disrupting ATP production and limiting the cell’s energy supply, leading to cellular dysfunction and increased susceptibility to stress [11].

**Fig. 1:**
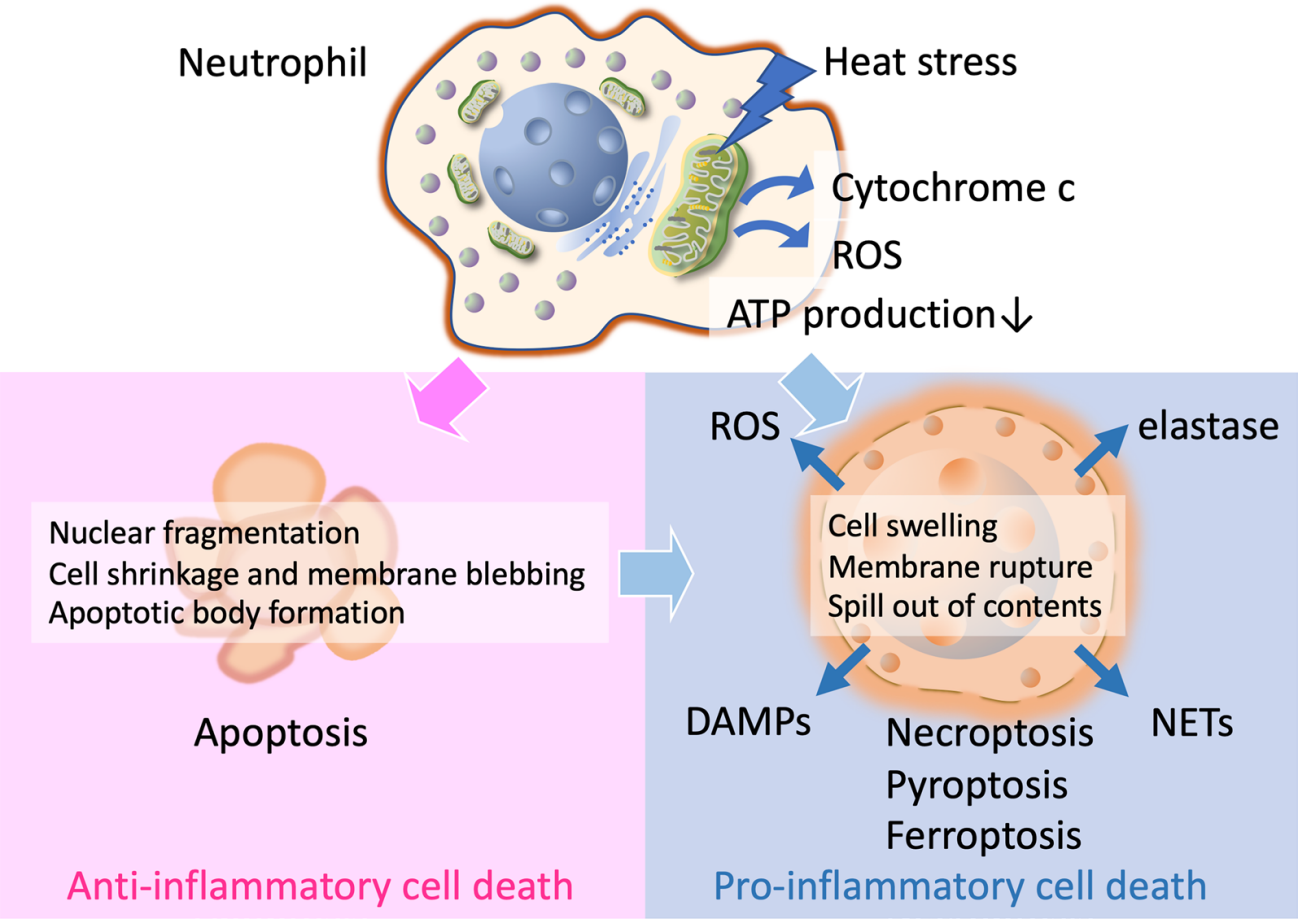
Pro-inflammatory and anti-inflammatory cell death mechanisms in heatstroke. Heat stress induces various types of programmed cell death. Apoptosis, which is anti-inflammatory, is characterized by nuclear fragmentation and cell shrinkage. Cells form apoptotic bodies that are cleared by phagocytes. In contrast, proinflammatory cell deaths, such as necroptosis, pyroptosis, and ferroptosis, share similar features, including cell swelling and ruptured nuclei. These cells release their cytoplasmic and nuclear contents, which trigger proinflammatory reactions

The damage to mitochondria induces apoptosis and necrosis [12]. At extreme temperatures (e.g., 43 °C or higher), hyperthermia induces apoptosis via mitochondrial pathways, such as releasing cytochrome c into the cytoplasm, leading to cell death. This mechanism is used therapeutically in hyperthermia-based cancer therapies to induce apoptosis in tumor cells [13]. However, since the apoptotic cell death pathway requires ATP, cells develop necrosis with the depletion of ATP, which ultimately induces inflammation [14] (Fig. 1). In summary, hyperthermia-induced mitochondrial dysfunction caused by increased membrane permeability, excess generation of ROS, and impaired respiratory chain reaction leads to apoptosis and necrosis, which results in multiple organ dysfunction. Thus, thermal injury of mitochondria is one of the essential mechanisms of death in heatstroke [15].

## Hyperthermia induces autophagy-related cell death

Another mechanism that can contribute to cellular damage is autophagy. While autophagy is primarily a system designed to protect cells from various types of damage, its effects can vary depending on the severity of stress [16]. Hyperthermia, for example, can cause protein misfolding and aggregation, triggering cellular stress responses such as the unfolded protein response and heat shock protein (HSP) expression. These stress responses often activate autophagy to clear misfolded proteins and maintain proteostasis [17]. Under moderate hyperthermia, autophagy generally acts as a protective mechanism, helping cells survive by removing damaged components and restoring cellular homeostasis [18]. However, in cases of severe or prolonged hyperthermia, heat stress‐mediated suppression of activation of autophagy and autophagosomal degradation, which may enable the persistence of damaged mitochondria and promote a dysfunctional intracellular environment [19]. This occurs when autophagy begins to degrade essential cellular components, resulting in dysfunction and, ultimately, cell death. In the context of severe heatstroke, overactivation of autophagy can exacerbate tissue damage. Autophagy may interact with both non-inflammatory apoptotic pathways and inflammatory processes associated with autophagic cell death, further amplifying cellular injury. Taken together, autophagy plays a dual role in heatstroke. It can protect cells by mitigating damage and maintaining homeostasis, but excessive or dysregulated autophagy can exacerbate cellular damage and cell death. The ultimate outcome depends on the balance between its protective and pathological effects.

## Hyperthermia induces cell death

**Fig. 2:**
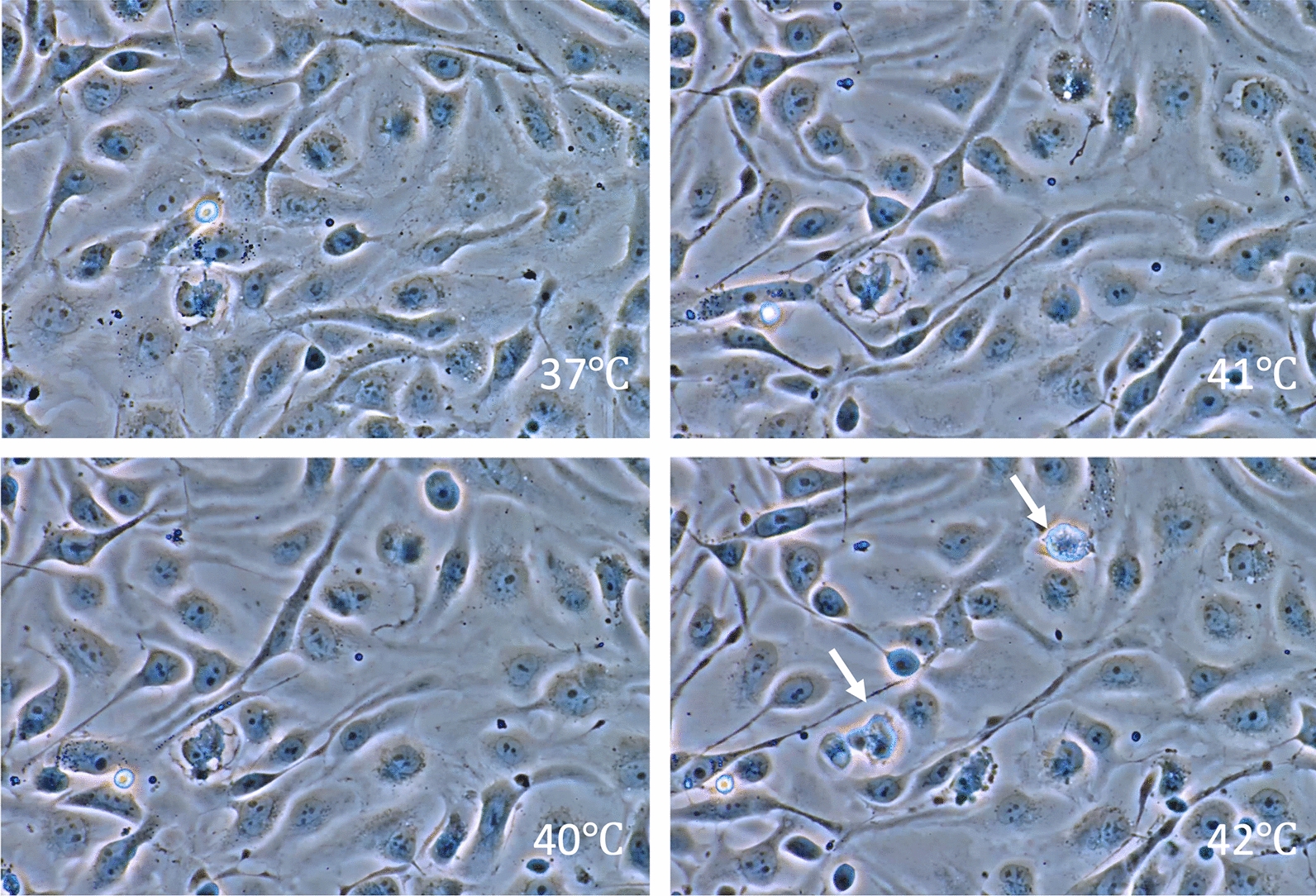
Endothelial cell damage. Phase-contrast images of cultured vascular endothelial cells exposed to heat from 37 to 42 °C showed that most cells maintained their shape, though some underwent cell death at 42 °C

**Fig. 3:**
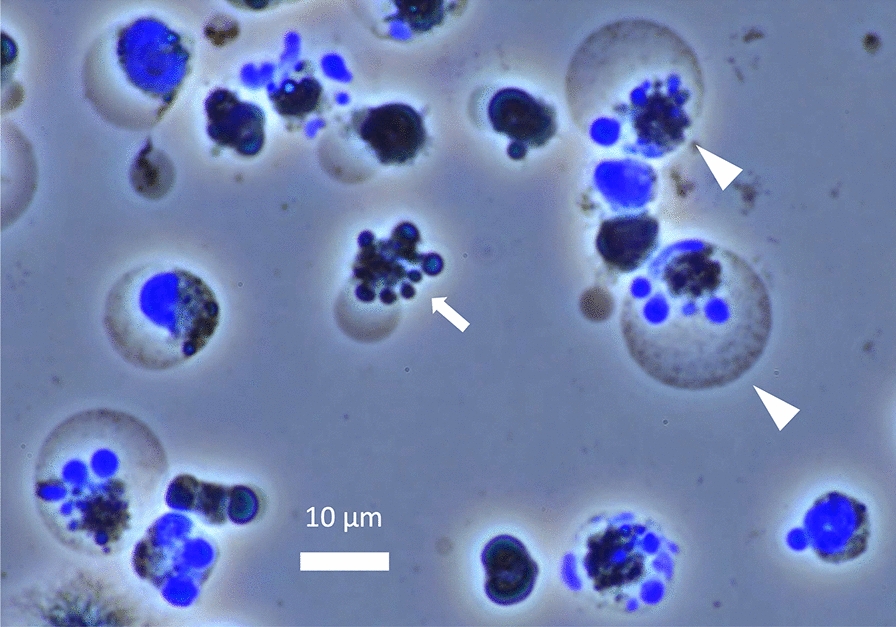
Leukocyte cell death induced by hyperthermia. Leukocytes obtained from rats were exposed to heat (42.0 °C) in vitro, and their morphological changes observed under a microscope using 4′,6-diamidino-2-phenylindole (DAPI) staining. Apoptotic cells showed cytoplasmic shrinkage and fragmentation (arrows), while necrotic cells exhibited ballooning (arrowheads)

**Fig. 4:**
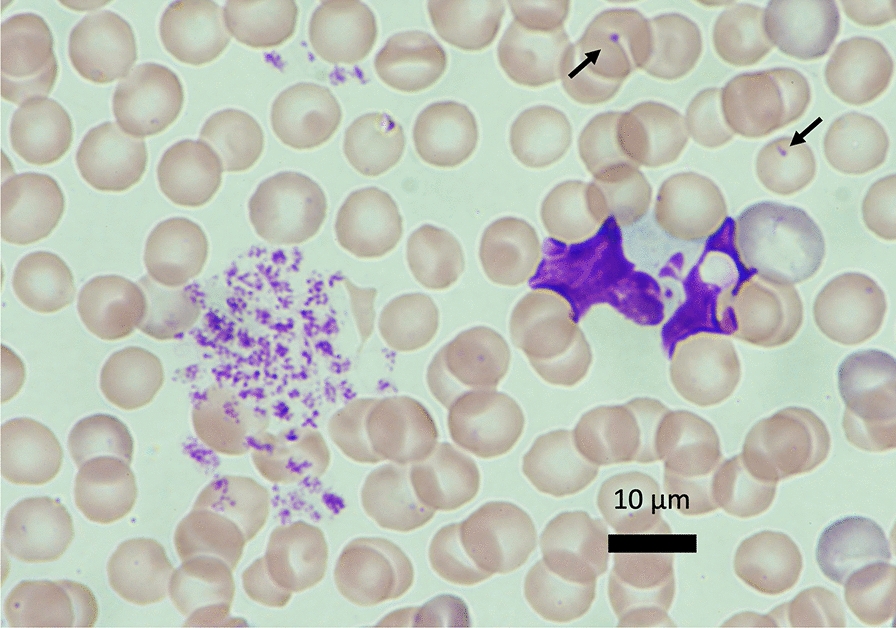
Leukocytes and platelets in heatstroke. The blood film obtained from a rat subjected to 41.5 °C was stained by May–Giemsa staining. The leukocytes were severely damaged, and nuclei were ruptured. Platelets were aggregated. Howell–Jolly bodies (arrows), nuclear remnants, are observed in red blood cells. Their presence may indicate increased red cell turnover

**Fig. 5:**
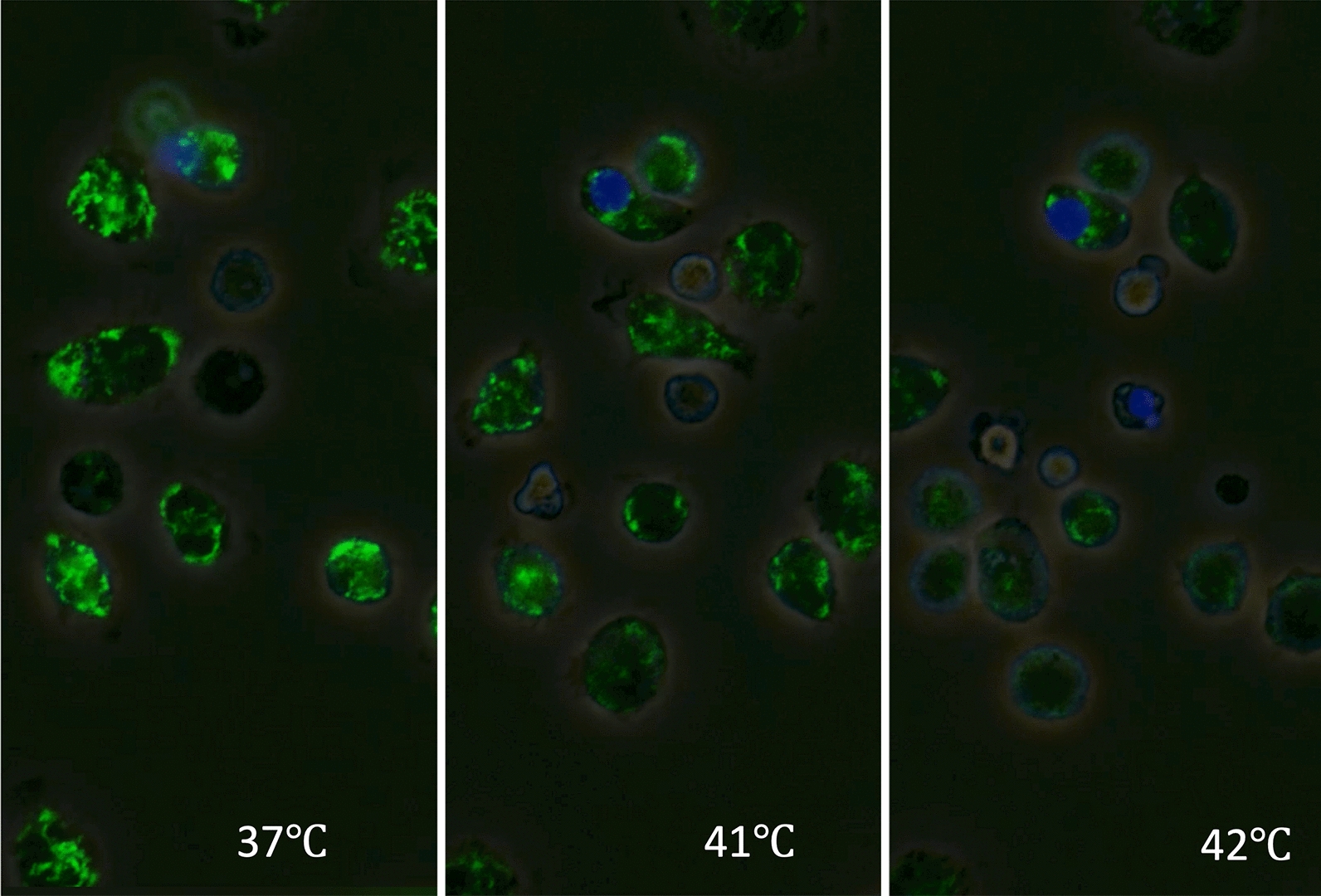
Mitochondrial damage under hyperthermia. Leukocytes obtained from rats were exposed to heat in vitro (42.0 °C). Mitochondria were stained using immunofluorescence [MitoBright LT ™, fluorescein isothiocyanate (FITC), green] and observed under a microscope. The intensity of mitochondrial staining diminished as the temperature increased, indicating a possible loss of mitochondrial integrity or function due to heat stress

The effects of hyperthermia have been studied as a cancer therapy [20]. Whole-body hyperthermia induces cell death through multiple mechanisms [21]. Depending on the temperature and duration of exposure, hyperthermia can trigger programmed (i.e., non-inflammatory) apoptosis or non-programmed and inflammatory necrosis. Cell viability differs between cell types, and blood cells are thought to be more susceptible to heat in general [22]. For example, cultured endothelial cells can survive under a temperature of 42 °C for 1 − 2 h (Fig. 2). In contrast, ballooned or ruptured leukocytes, along with aggregated platelets, were observed in blood smears from rats when their body temperature reached 41.5 °C [23] (Figs. 3, 4). In vitro, moderate hyperthermia of 40–41.5 °C for 1 h suppresses the movement of leukocytes and induces cell death through mitochondrial damage (Fig. 5). Human platelet aggregation induces apoptosis, leading to subsequent activation of the caspase pathway [24]. Extreme hyperthermia around 43 °C or prolonged duration leads to necrosis by causing protein denaturation and membrane instability [25]. Hyperthermia compromises mitochondrial membrane integrity, leading to increased membrane permeability, membrane potential loss, and cytochrome c release, which are hallmarks of apoptosis initiation [13]. At the same time, elevated temperatures affect cellular structures, such as the cytoskeleton and plasma membrane, causing protein unfolding and aggregation [26]. Red blood cell turnover is accelerated, and an increased count of nucleated red blood cells in the peripheral blood is associated with heatstroke severity, making them a useful prognostic marker for mortality risk [27]. The above-mentioned structural instability interrupts essential cellular processes and contributes to cell death [28].

Serum levels of HSPs, particularly HSP70, reflect cellular stress responses in heatstroke [29]. HSPs, are useful indicators of cellular heat tolerance and can serve as potential prognostic markers for recovery and survival [30]. They are produced in response to heat stress and help protect cells by stabilizing proteins, repairing damaged proteins, and preventing apoptosis. Their levels often correlate with the degree of cellular stress and the ability of cells to recover from thermal injury [31]. Overall, hyperthermia causes various types of cell death, such as anti-inflammatory apoptosis, pro-inflammatory pyroptosis, necroptosis, and. ferroptosis; these modes of cell death regulate heat stress and further development of heatstroke at different levels [5]. In the case of heatstroke, cell death is caused primarily by mitochondrial dysfunction; but, in extreme hyperthermia, necrotic cell death is induced through protein denaturation, DNA damage, and structural disruptions. It should be noted that cell death styles differ depending on cell types, cell aging, and other conditions.

## Effects of hyperthermia on inflammation

Whole-body hyperthermia has notable effects on inflammation, influencing immune responses in ways that can be both beneficial and detrimental. Increased body temperature has been shown to amplify proinflammatory responses against infection by increasing the production of cytokines like tumor necrosis factor-α (TNF-α) and interleukin-1 (IL-1α) [20]. Although such reactions help antitumor effects and benefit concurring infection, excess high body temperature seen in heatstroke may be disadvantageous. Heat-induced host responses result in dysregulated inflammation, hyper-coagulation, and increased danger signals [32]. Schleder et al. [33] reported high mobility group box protein 1 (HMGB1), histone H3, HSP72, and IL-1α as biomarkers reflecting the severity of heatstroke. High body temperature also increases the activity and recruitment of immune cells, such as monocytes and neutrophils, to inflamed tissues. In animal models, febrile-range hyperthermia (39 °C to 40 °C) increased proinflammatory chemokine production and neutrophil infiltration, which are advantageous for host defense, but also known to intensify inflammatory lung injury [34].

Hyperthermia has been known to exhibit immunomodulatory effects by enhancing the release of anti-inflammatory cytokines, such as IL-10, which helps to counterbalance inflammation [35]. This dual response suggests hyperthermia can support immune modulation in certain contexts, potentially reducing excessive inflammation [36]. Hyperthermia can also block the activation of proinflammatory signaling pathways, such as nuclear factor κB (NF-κB) and mitogen-activated protein kinase (MAPK) [37]. Short-term hyperthermia also inhibits NF-κB translocation in fibroblast-like synoviocytes to reduce proinflammatory gene expression, suggesting that hyperthermia could serve as a potential therapy for inflammation-related diseases [38]. Thus, hyperthermia can amplify and modulate inflammation by enhancing immune cell recruitment and cytokine release, while suppressing proinflammatory signaling, depending on the context. This makes hyperthermia both a supportive yet complex factor in managing inflammation.

## Effects of hyperthermia on coagulation

Dysregulated inflammation and coagulation are primary factors involved in organ dysfunction under various insults [39]. Hyperthermia affects coagulation processes in several ways, often creating a prothrombotic state [40]. Hyperthermia induces dynamic changes in coagulation and platelet function [41]. First, the coagulation system and platelet aggregation are stimulated by activated inflammation, leading to prothrombotic conditions. However, this prothrombotic state turns into a coagulopathic state if the hyperthermia is extreme or prolonged. Diehl et al. [42] reported significant thrombocytopenia and prolonged clotting times in an extreme hyperthermia model of canines (42.5 °C for 90 min).

Severe hyperthermia, such as heatstroke, can lead to disseminated intravascular coagulation (DIC). DIC is represented by activated coagulation with systemic thrombosis within the microvasculature, followed by impaired hemostasis due to the depletion of clotting factors and platelets [43]. This phenomenon has been noted both in therapeutic hyperthermia and environmental heat exposure [44]. Hyperthermia affects the activity of coagulation factors and clotting times, such as activated partial thromboplastin time (aPTT) and prothrombin time (PT), which prolong significantly when the temperature reaches 43 °C [45]. Other than the direct effect, hyperthermia can indirectly affect coagulation by impairing hepatic function, a critical source for using clotting factor synthesis to exacerbate coagulopathy during extreme heat exposure [46]. Recent studies suggested the usefulness of viscoelastic tests such as thromboelastography (TEG) and rotational thromboelastometry (ROTEM) in predicting outcomes of heatstroke [47, 48]. Overall, hyperthermia creates a complex coagulation imbalance that can increase thrombotic risks in mild cases or lead to DIC and bleeding complications in severe cases [49].

## Hyperthermia and organ function

The development of multiorgan dysfunction is a major cause of death in heatstroke. Direct heat damage, excess inflammation, oxidative stress, cell death, coagulation disorders, and gastrointestinal microbial translocation are the factors involved in pathogenesis [50]. Heatstroke can trigger systemic inflammation, coagulopathy, rhabdomyolysis, cerebral edema, pulmonary edema, heart failure, and renal and hepatic dysfunction [51]. Hyperthermia increases oxidative stress in the liver, leading to mitochondrial damage and the accumulation of toxic byproducts like malondialdehyde. In animal models, sustained hyperthermia impairs hepatic function by altering cell structure and reducing antioxidant defenses, making the liver more vulnerable to subsequent infection and injury [52].

In renal dysfunction, hyperthermia exacerbates tissue malcirculation and ischemic injury by thromboinflammation, leading to acute kidney injury (AKI) similar to mechanisms that occur in sepsis [4, 53]. The high temperatures affect mitochondrial ATP production and the integrity of the cytoplasmic membrane, leading to increased cell death in kidney tissues and overall renal dysfunction [54]. Urinary markers such as neutrophil gelatinase-associated lipocalin (NGAL), kidney injury molecule-1 (KIM-1), and liver fatty acid-binding protein (L-FABP) are effective in identifying AKI due to heatstroke. These biomarkers correlate with heat-related illness severity and are valuable for early AKI detection [55].

Hyperthermia stresses the cardiovascular system, increasing heart rate and blood pressure, due to physiologic compensation to increase cutaneous circulation facilitating heat dissipation to lower body temperature. Subsequently, baroreflex control appears to be impaired, potentially due to a diminished vasoconstrictor response in the cutaneous circulation [56]. Extreme hyperthermia causes myocardial dysfunction and heart failure, and in animal models causing acute hypotension at 43 °C [40]. The sudden onset of myocardial injury is likely induced by mitochondrial dysfunction, imbalance production of ROS, and oxidative damage [57]. Hyperthermia also leads to endothelial dysfunction, which affects blood flow and can reduce perfusion to organs like the intestines [58].

The central nervous system is particularly vulnerable to hyperthermia. Heatstroke is typically characterized by the rapid rise of core body temperature above 40 °C and central nervous system dysfunction, leading to short-term neurological dysfunction and longer-lasting cognitive deficits [59]. Severe hyperthermia can cause inflammation and damage to neurons, with the cerebellum and hypothalamus being especially sensitive to heat, increasing the risk of long-term neurological issues [60].

## Genomic predispositions and severity of heatstroke

Genomic predispositions are key factors influencing susceptibility to heatstroke and exertional heat illness. Variants in specific genes, such as ASPH, which encodes junctin (a regulator of excitation–contraction coupling), have been linked to exertional heat illness and malignant hyperthermia susceptibility. Rare, pathogenic heterozygous variants in the ASPH gene may disrupt calcium regulation, increasing the risk of heat-related illnesses [61]. Similarly, mutations in the RYR1 gene, which encodes the type I skeletal muscle ryanodine receptor, are a well-established genetic cause of malignant hyperthermia susceptibility and have also been implicated in exertional heat illness. These mutations likely contribute to abnormal calcium handling in muscle cells under heat stress [61].

Genes encoding HSPs, such as HSPA1B, HSP90AA2, and DNAJA1, have also been studied. Single nucleotide polymorphisms (SNPs) in these genes are thought to influence the ability to manage cellular stress caused by heat, affecting an individual’s heat tolerance and risk of exertional heat illness [62].

In addition to genetic factors, epigenetic modifications such as DNA methylation and histone modifications play a role in the severity of heatstroke. These modifications can influence how individuals respond to heat stress by creating a “molecular memory” of past environmental exposures, which may alter future heat tolerance [63]. Understanding these genomic and epigenetic factors is essential for identifying individuals at higher risk and developing targeted prevention strategies.

## Effects of hypothermia on mitochondria and whole-body

Hypothermia leads to death through complex mechanisms affecting the cardiovascular, neurological, and metabolic systems. As core body temperature drops, the heart becomes susceptible to arrhythmias, particularly ventricular fibrillation. This increased susceptibility is further exacerbated by decreased temperature, causing bradycardia and cardiac arrest [64]. These responses are due to metabolic and electrolyte disturbances. Hypothermia slows enzymatic reactions, leading to metabolic acidosis. Concurrently, electrolyte imbalances, particularly potassium shifts, are induced, destabilizing cardiac cells and increasing the risk of fatal arrhythmias [65].

However, hypothermia has notable cellular protective effects via mitochondrial protection, especially during conditions of cellular stress such as ischemia or oxidative damage. Studies on hypothermic treatment in cardiac cells show that it reduces mitochondrial permeability transition pore (mPTP) opening, preventing cytochrome c release and thereby reducing apoptosis. This effect is associated with improved cellular viability and reduced ischemic damage [66]. Furthermore, hypothermia decreases ROS production in mitochondria during stress conditions [67]. For instance, in models of cardiac and cerebral ischemia, hypothermia has been shown to limit oxidative stress by inhibiting ROS generation, preserving mitochondrial complexes, and supporting enzyme functions that are essential for energy production [68]. Eventually, hypothermia suppresses the energy demand, which helps to preserve ATP levels and protect mitochondria [69].

The protective effects of hypothermia on mitochondria suppress apoptosis, particularly after traumatic brain injury or ischemic events, by decreasing the activation of apoptotic proteins like caspase-3 [70]. This protective mechanism is crucial for minimizing cell death in tissues like the myocardium following oxygen-deprivation events [71].

## Effects of hypothermia on inflammation and coagulation

Hypothermia has suppressive effects on inflammation to maintain homeostasis. First, hypothermia lowers proinflammatory cytokine levels, such as IL-1β and TNF-αreducing production by microglial cells preventing excessive inflammation and injury following brain injury [72]. Second, hypothermia can increase anti-inflammatory cytokine IL-10 levels while decreasing nitric oxide (NO) production to minimize inflammation without overly suppressing immune defense [73]. Moderate hypothermia has been shown to decrease leukocyte rolling and adhesion in blood vessels, reducing immune cell infiltration into tissues and lowering tissue inflammation. This effect is linked to lower expression of adhesion molecules such as intracellular adhesion molecule-1(ICAM-1) on endothelial cells [74]. These findings are primarily derived from studies on controlled hypothermia and may not be fully applicable to cases of accidental hypothermia.

In contrast to beneficial effects on inflammation, hypothermia impairs the hemostasis that can increase bleeding in various clinical settings by inhibiting enzymatic reactions of coagulation, and clinically prolong PT and aPTT and clot formation [75]. Hypothermia also impairs platelet function, affecting their ability to adhere and aggregate effectively, critical steps in clot formation. Decreased platelet function is observed at lower temperatures, leading to compromised hemostasis [76]. Sustained hypothermia may promote microvascular thrombosis despite impairing coagulation and platelet functions. Animal studies show increased microvascular thrombus formation due to hypothermia-induced platelet aggregation, which may contribute to microvascular complications [77]. This enhanced platelet aggregation also occurs in conditioned hypothermia, prompting consideration for antiplatelet agents in patients [78].

## Treatments for hyperthermia

Effective heatstroke treatments are summarized as rapid cooling and supportive care to prevent multiorgan dysfunction. In the following part, we introduce cooling and other trials.

### Standard treatments

Cooling is preferably performed with cold water immersion, along with hemodynamic stabilization. Especially for severe cases of exertional heatstroke, cold water immersion in the bath tab is considered the gold standard for reducing core body temperature [79, 80]. When cold water immersion is not feasible, alternative methods such as cooling blankets, evaporative cooling (spraying water on the skin and using fans), and conductive cooling with ice packs applied to areas with high vascularity are employed [81]. In cases of severe heatstroke, internal cooling, such as gastric lavage and cold hemodialysis with iced saline, has demonstrated efficacy [82]. In severe cases with neurological involvement, therapeutic hypothermia (cooling to 33 °C) has shown potential in preventing neurological sequelae. External cooling devices are used clinically to achieve and maintain mild hypothermia to reduce brain inflammation and injury in the early recovery phase [83, 84]. Managing hemodynamic stability is essential in heatstroke due to distributive shock and hypovolemia risk. Continuous monitoring and fluid replacement are critical for maintaining blood pressure and circulation, especially in critical care settings [85].

### Expected treatments

#### Antioxidants

Agents like vitamin C, vitamin E, or N-acetylcysteine (NAC) may help reduce oxidative stress and protect against cellular damage in heatstroke. Peng et al. [58] reported that coagulation abnormalities in heatstroke rats caused by endothelial glycocalyx damage were improved by NAC (*N*-acetylcysteine) through its protective effect on the glycocalyx by preventing the generation of ROS.

#### Molecular hydrogen

Studies have explored the use of hydrogen gas for its antioxidant properties, potentially reducing organ damage from oxidative stress. Truong et al. [86] demonstrated that 2% hydrogen gas significantly improved survival in heatstroke rats and partially preserved the thickness of the endothelial glycocalyx. Additionally, serum levels of endotoxin, syndecan-1, malondialdehyde, and TNF-α decreased while superoxide dismutase levels increased. These findings suggest that inhaling 2% hydrogen may mitigate damage to the vascular endothelial glycocalyx through its antioxidative and anti-inflammatory effects.

#### Heat shock proteins

HSPs play a critical role in protecting cells from heat-induced damage. Therapies aimed at boosting HSP expression could enhance cellular tolerance to heat stress, and HSP-inducing agents (e.g., geranylgeranylacetone) are being explored to protect cells and enhance recovery. Zhao et al. [87] reported that geranylgeranylacetone pretreatment significantly suppressed heat-induced damage. Geranylgeranylacetone preconditioning increased plasma and brain levels of IL-10 and HSP70 in models of heatstroke.

#### Mitochondria protection

Heat stress can lead to mitochondrial dysfunction, resulting in cell death. Agents that protect or stabilize mitochondria, such as MitoQ, an antioxidant targeting mitochondria, are being investigated for their potential to reduce heat-induced cellular damage. Mayorga et al. [88] reported that heatstroke-induced alterations in animal performance, inflammation, and metabolism were partially ameliorated by orally administered MitoQ.

## Treatments for hypothermia

Accidental hypothermia management requires rapid assessment and tailored rewarming strategies to prevent further complications and improve survival. Successful management of accidental hypothermia relies on clinical monitoring and support of cardiopulmonary function and metabolic derangements [89]. For moderate-to-severe hypothermia (< 30 °C), active core rewarming, including options like heated intravenous fluids, warmed humidified oxygen, and extracorporeal membrane oxygenation (ECMO), is preferred. ECMO and other extracorporeal life support techniques provide continuous rewarming and are especially effective in cases of hypothermic cardiac arrest [6]. In patients, to avoid cardiopulmonary arrest, hemodynamic resuscitation and/or stabilization is crucial, along with correcting metabolic derangements, including acidosis and coagulopathy [90].

## Research perspectives

Breakthrough research is necessary to develop novel treatments for heatstroke. Focusing on energy depletion mechanisms and cellular resilience provides a promising approach [5, 91]. This strategy could improve the understanding of heat-induced cell damage and lead to targeted therapies [92]. In addition, the combination of the physiological signs and biomarker-oriented evaluation is necessary for correct risk stratification. This could aid in identifying at-risk individuals and customizing interventions [93].

As for hypothermia, mild-to-moderate hypothermia continues to be studied in clinical trials for its neuroprotective effects in conditions like stroke and traumatic brain injury [94]. Research is focused on understanding how cooling influences inflammation, oxidative stress, and cell survival while also optimizing protocols for cooling duration, temperature, and rewarming rates [95]. Future studies should aim to elucidate the molecular signaling pathways and proteins activated by cold temperatures, including cold shock proteins and hypothermia-induced pathways [96]. These investigations will enhance the neuroprotective effects of hypothermia and improve treatment outcomes in both acute and chronic neurological conditions.

## Conclusion

Extreme temperatures significantly impact health. Hyperthermia, particularly above 40 °C, imposes intense stress on the body via mitochondrial damage, inflammatory responses, and ultimately cell death. Conversely, hypothermia generally offers cytoprotective effects, suppressing cellular metabolism and preserving mitochondrial function. With climate change likely to intensify, it is essential to prepare for temperature-related health challenges.

## Data Availability

Not applicable.
